# Beef production in integrated crop–livestock systems of Uruguay: assessing economic, environmental, and food security trade-offs

**DOI:** 10.1093/af/vfag027

**Published:** 2026-06-05

**Authors:** Fabiana Pereyra-Goday, Walter Ayala, Jesús Castillo, Asma Jebari, Graham A McAuliffe, Michael R F Lee, Pablo Rovira, Taro Takahashi, José A Terra, M Jordana Rivero

**Affiliations:** Instituto Nacional de Investigación Agropecuaria, Estación Experimental del Este, Treinta y Tres, Uruguay; Instituto Nacional de Investigación Agropecuaria, Estación Experimental del Este, Treinta y Tres, Uruguay; Instituto Nacional de Investigación Agropecuaria, Estación Experimental del Este, Treinta y Tres, Uruguay; Rothamstd Research, North Wyke, Devon, UK; Harper Adams University, Newport, Shropshire, UK; Harper Adams University, Newport, Shropshire, UK; Instituto Nacional de Investigación Agropecuaria, Estación Experimental del Este, Treinta y Tres, Uruguay; Agri-Food and Biosciences Institute, Hillsborough, Co Down, UK; Instituto Nacional de Investigación Agropecuaria, Estación Experimental del Este, Treinta y Tres, Uruguay; Rothamstd Research, North Wyke, Devon, UK

**Keywords:** carbon footprint, crop-pasture rotations, mixed farming systems, nitrogen use efficiency, sustainable intensification

Implications

- Multicriteria evaluation of integrated crop–livestock systems is essential to reveal trade-offs among productivity, economic returns, food security (quantity vs. nutritional quality of protein), and environmental impacts (erosion, emissions, nitrogen use efficiency).
- Long-term experiments play a key role in supporting the development of evidence-based policy, land-use regulations, and producer decisions in agricultural-based regions.
- Our results highlight the importance of sustainable intensification of production systems through the combination of pastures and crops and their impact on productivity, environmental aspects, and long-term sustainability.

## Introduction

By 2034, the world’s population is projected to grow by 0.8% per year, while demand for agricultural products is expected to increase to a greater extent, driven primarily by demographic trends rather than per capita consumption (Organisation for Economic Co-operation and Development/Food and Agriculture Organisation, 2025). This widening gap presents both opportunities and challenges for producers, particularly in agricultural export-dependent countries such as Uruguay, where beef, soybeans, dairy products, and rice account for nearly half of total exports.

Sustainable intensification seeks to raise yields without expanding agricultural land, while safeguarding ecosystem health and reducing the environmental footprint of food production (Campanhola and Pandey, 2019; Ajibade et al., 2023) and is vital for achieving global food security, improving resource-use efficiency, and ensuring the long-term viability of agricultural systems (Semmartin et al., 2023). It promotes environmentally sound practices, reduced reliance on external inputs, and greater resilience to climatic and economic variability (Arístide et al., 2020). Key strategies of sustainable intensification include ecological intensification, conservation agriculture, and integrated crop–livestock systems (ICLS) (Cortner et al., 2019). Integrated crop–livestock systems are considered one of the most promising models for achieving sustainable intensification, particularly in temperate regions, because they can simultaneously improve productivity, resource-use efficiency, soil health, and resilience to climate and market variability (Campanhola and Pandey, 2019; Cortner et al., 2019; Ajibade et al., 2023).

Integrated crop–livestock systems produce most of the world’s milk and ruminant meat and are particularly important for the livelihoods and food security of poor people in developing countries. They occur in nearly all agro-ecological zones in developing countries under widely disparate climatic and soil conditions. Mixed systems are particularly important for food security, providing most of the staples consumed by the world’s poor: between 41% and 86%of the maize, rice, sorghum, millet, 75% of the milk, and 60% of the meat (Herrero et al., 2010).

As pressure grows to produce food more sustainably, evaluation of ICLS performance, encompassing productive, economic, social, and environmental dimensions, has become essential (Lee et al., 2021). Indicators such as nitrogen use efficiency, greenhouse gas emissions, and total (crop + livestock) productivity per unit of input simultaneously address sustainable livestock transformation, food security, and environmental impacts (Paruelo and Sierra, 2023). Researching ICLS is challenging owing to the large land areas required, high costs, labor demands, and decision-making complexity. Long-term experiments (LTEs) at the systems scale provide a valuable framework, replicating real-world conditions and capturing processes that smaller-scale trials cannot (Pereyra-Goday et al., 2022).

Here, we adopt an integrative assessment approach to explore trade-offs among productivity, environmental outcomes, and food-security metrics across contrasting ICLS in Uruguay. This country offers a highly relevant test case for temperate ICLS globally. Characterized by widespread no-till crop–pasture rotations (García-Préchac et al., 2004), Uruguay has decades of experience with these systems under commercial conditions. Findings from Uruguayan LTEs therefore provide valuable insights into the real-world performance and trade-offs of ICLS that are directly applicable to other temperate mixed-farming regions facing similar pressures regarding sustainable intensification, food versus feed competition, and multifunctional land use. These systems help control soil erosion and degradation and deliver multiple benefits: increased soil carbon content, improved crop yields following the pasture phase, enhanced pest and disease control, greater soil microbial biomass, reduced use of inorganic fertilizer, and improved nutrient-use efficiency (Terra et al., 2006; De Faccio Carvalho et al., 2010; Ward et al., 2016; Rubio et al., 2021; Cerecetto et al., 2025).

By combining productivity and environmental indicators with economic and social outcomes, while purposefully avoiding the identification of a single optimal production system, this study offers insights into the capacity of ICLS to meet food-security objectives sustainably. We synthesize a wide range of results reported elsewhere and examine how these systems can reduce greenhouse gas emissions (Pereyra-Goday et al., 2024), improve nitrogen use efficiency (Pereyra-Goday et al., 2025), and maintain productive capacity (Pereyra-Goday et al., 2022), thereby enhancing food security while preserving environmental quality, maintaining soil health, and reducing feed-food competition in the context of resource use intensification. This multidimensional approach supports the development of sustainable intensification strategies and provides a framework for balancing productivity with environmental stewardship in Uruguay and similar agricultural regions. Finally, we highlight the relevance of this LTE in the national context and summarize its principal contributions from a broad, integrative perspective.

## The Palo a Pique Long-Term Experiment and Its Rich Datasets

The Palo a Pique (INIA, Uruguay) LTE includes four production systems (three pasture-crop rotation systems and a forage rotation), offering different temporal and spatial combinations of land use. Table 1 outlines the crops in each rotation (either pasture-crop or pasture-only rotation) and the purpose of each phase (crop production or grazing). Although the experiment originated in 1995, the data reported in this study correspond to those obtained from the redesign implemented in 2019 (Rovira et al., 2020), when a customized livestock management strategy was introduced in each system. The study evaluated four production systems based on 3 years of data (2019–2022). Details pertaining to the management of each system can be found in Pereyra-Goday et al. (2022).

**Table 1.** Pasture and crop sequence for each rotation at Palo a Pique long-term experiment, Treinta y Tres, Uruguay (Pereyra-Goday et al., 2022)

System*^a^*	Purpose of crop phase	Rotational year					
		Year 1	Year 2	Year 3	Year 4*^b^*	Year 5*^b^*	Year 6*^b^*
CC	Grain	Wheat/Soybean	Oat/Sorghum				
	Grazing	Oat/Sorghum	Ryegrass/Foxtail millet				
SR	Grain	Oat/Sorghum	Black Oat/Soybean	Wheat + P1*^b^* ^,^ *^c^*	P2*^c^*		
	Grazing	Idem CC	Idem CC	P1	P2		
LR	Grain	Oat/Sorghum	Black Oat/Soybean	Wheat + P1*^c^*	P2*^c^*	P3*^c^*	P4*^c^*
	Grazing	Idem CC and SR	Idem CC and SR	P1	P2	P3	P4
FR	Grazing	Fescue	Fescue	Fescue	Fescue	Fescue	Fescue

a CC: Continuous Cropping; SR: Short Rotation; LR: Long Rotation; FR: Forage Rotation.

b Pasture follows by the age of the pasture (1 to 2 in SR and 1 to 4 in LR).

c Pastures in the crop area (in SR and LR) are grazed.

Economic data were collected on variable costs and revenues from grain and livestock sales based on the physical inputs and outputs presented in Pereyra-Goday et al. (2022). The contribution to food security through the production of human-edible energy (HEE) and human-edible protein (HEP) was calculated using the methodology proposed by Mosnier et al. (2021) for both grain and meat production. The quality-adjusted digestible indispensable amino acid (DIAA) supply (kg/ha) was then estimated by multiplying the HEP yield of each crop by its respective Digestible Indispensable Amino Acid Score (DIAAS) value divided by 100 (i.e., 111.6 for meat, 99.6 for soya, 40.4 for wheat (Ertl et al., 2016) and 67 for oats (Abelilla et al., 2018) and summing the contributions from each component (crop and livestock) to provide an integrated measure of corrected nutritionally effective protein output per hectare. Soil erosion was estimated using the Universal Soil Loss Equation (USLE-RUSLE) model (software Erosión 6.0, MGAP, Uruguay), a tool used by environmental regulators in Uruguay to assess a farm’s compliance with land management policy (Pérez-Bidegain et al., 2018). Detailed description of the methodology and coefficients can be found in Pereyra-Goday et al. (2024).

## Key Findings and Sustainability Trade-Offs

**Figure 1.**
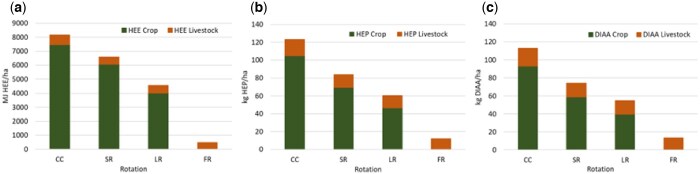
Contribution to food security across the four systems: Human edible energy (HEE) (a), Human edible protein (HEP) (b), and quality-adjusted digestible indispensable amino acid (DIAA) supply (c). Values are 3-year average (2019–2022) at the Palo a Pique long-term experiment, Uruguay: Continuous cropping (CC) maximizes quantity-based food output, while systems with longer pasture phases shift toward higher nutritional quality from animal protein. Short rotation (SR), long rotation (LR), and forage rotation (FR).

This LTE demonstrates clear patterns and inherent trade-offs in the sustainability of ICLS. Systems with longer pasture phases, particularly the long rotation, provided the best overall balance across economic, environmental, and soil conservation indicators. These systems delivered higher whole-system gross margins, improved nitrogen use efficiency through biological fixation by legumes, and soil erosion rates well below the regulatory tolerance limit of 7 Mg/ha/year (Pereyra-Goday et al., 2024, 2025) (Table 2). In contrast, the continuous cropping system, which lacks a perennial pasture phase, achieved the highest short-term contributions to food security, delivering the greatest HEE, HEP, and DIAA supply per hectare (Figure 1). However, this intensive cropping strategy resulted in soil losses exceeding the tolerance threshold (7.16 Mg/ha/year) and the highest associated economic costs, posing significant long-term sustainability risks (Pravia et al., 2019; Pereyra-Goday et al., 2022).

**Table 2.** Main indicators calculated for the evaluated systems: continuous cropping (CC), short rotation (SR), long rotation (LR), and forage rotation (FR)

Parameter	Unit	CC	SR	LR	FR
Liveweight production	kg LW/ha	426 **Ρ**	418 **Ρ**	369 **□**	310 **□**
Crop production (soybean/wheat/oat/sorghum)	t/ha	2.4/2.21/-/4.97 **□**	2.48/2.68/1.81/5.65 **Ρ**	2.76/2.59/2.18/5.29 **Ρ**	–
Forage production	kg DM/ha	5206 **□**	5763 **Ρ**	5399 **□**	6867 **Ρ**
GHG emissions intensity	kg CO_2_-eq/kg LW	11.3	11.8	11.8	16.4
GHG emissions intensity	kg CO_2_-eq/ha	2795	2734	2727	2607
GHG emissions intensity	kg CO_2_-eq/kg soybean/wheat/oat/sorghum	1.36/0.61/-	1.24/0.54/0.57	1.01/0.43/0.47	–
Nitrogen use efficiency (crop)	%	62.5 **Ρ**	83.8 **Ρ**	77.5 **Ρ**	–
Nitrogen use efficiently (livestock)	%	24.4 **□**	9.9 **Τ**	14.3 **Ρ**	5.5 **Τ**
Gross margin	US dollar/ha	181	200	285	197
Emissions per US dollar	kg CO_2_/US dollar	15.4	13.7	9.6	13.2
Human Edible Protein & Human Edible Energy	kg/ha	High	Medium	Medium	Low
Soil losses	t/ha	7.16 **Τ**	4.11 Ρ	3.17 **Ρ**	2.49 **Ρ**

Values outside the established optimal limits are marked in red/**Τ**symbol, intermediate values in yellow/**□** symbol, and optimal values in green/**Ρ** symbol (see Pereyra Goday, 2024). Indicators with no significant differences or without statistical analysis are shown in grey/no symbol. GHG: greenhouse gas.

Systems with intermediate pasture phases (short rotation) and forage rotation offered different compromises. Short rotation performed competitively in economic returns, while the forage rotation showed the lowest soil erosion. A particularly innovative and decision-relevant metric in this study is the emission intensity per US$ of gross margin. This indicator integrates economic performance with environmental impact and revealed that the long rotation was the most eco-efficient system (9.6 kg CO_2_-eq per US$ gross margin) (Table 2), followed by the short rotation, even when livestock strategies varied in intensity (Pereyra-Goday et al., 2024).

Regarding food security, systems with greater cropping intensity (continuous cropping and short rotation) produced more total HEE and HEP per hectare due to higher grain output. However, systems relying more on pastures reduced competition with human-edible feed and increased the relative contribution of high-quality animal protein, as evidenced by the more favorable DIAAS profile of beef (Wilkinson and Lee, 2018; Mosnier et al., 2021; McAuliffe et al., 2023) (Figure 1). This highlights the complementary role of livestock integration in nutritional outcomes.

Overall, these findings underscore that diversified ICLS with extended pasture phases (especially long rotations) better reconcile productivity, profitability, environmental performance, and soil conservation than highly specialized systems. Such patterns provide robust evidence to support policy and management decisions favoring sustainable intensification through well-designed crop–livestock integration in Uruguay and similar regions.

## Contributions of Palo a Pique Long-Term Experiment

Thirty years after the establishment of the Palo a Pique LTE, several phases have unfolded in parallel with technological developments, advances in research, and emerging challenges linked to the sustainable transformation of production systems. These dynamics have generated new research questions and evolving production objectives. Continuous support from practitioners, researchers, and technical advisors in decision-making has been essential to ensure that the experiment remains closely aligned with commercial production systems.

Beyond enabling the calculation of indicators and technical coefficients used as benchmarks by producers and technical advisors, the Palo a Pique LTE functions as a close reflection of commercial farming systems. This is possible due to its semicommercial scale and the implementation of crop rotations and livestock strategies that are representative of those used in practice for these soil types, serving as a “hub” for the relevant region where “spokes,” the producers, adopt and scale these interventions, which is part of the Global Farm Platform modus operandi (www.globalfarmplatform.org).

For policy design, the results reinforce the importance of maintaining ICLS within agricultural landscapes, not only for productivity gains but also for their contribution to soil conservation, nitrogen use efficiency, and long-term sustainability. As highlighted by Rovira et al. (2020), several outcomes of the Palo a Pique LTE have also contributed to public policy development. For example, soil erosion results, validated in Uruguay using datasets that included those generated in the experiment’s runoff plots, provided critical information for the design of the Soil Use and Management Plan (Pérez-Bidegain et al., 2018). Under national regulations, every crop producer must submit a land use and management plan demonstrating that the proposed rotation will result in an average annual erosion rate below the officially established threshold.

In the context of internationally recognized sustainability assessment and benchmarking frameworks applied to agri-food systems, the evidence generated by this long-term experiment provides a robust empirical basis to inform initiatives seeking to balance productivity, environmental integrity, and food security objectives. From an applied perspective, these findings highlight the opportunity, for example, for sustainability certification schemes to move beyond single indicator approaches and adopt multicriteria frameworks capable of more accurately capturing the performance of integrated systems, while explicitly recognizing context-specific trade-offs among productivity and environmental performance.

Taken together, the Palo a Pique LTE stands as a valuable platform where scientific evidence, commercial relevance, and policy-oriented insights converge, providing a robust foundation for advancing sustainability assessment and decision-making frameworks for ICLS operating under real-world constraints. LTEs are strategic research platforms for assessing the cumulative impacts of crop rotation and soil management practices on agroecosystems (Johnston and Poulton, 2018). Beyond generating long-term datasets, this ICLS LTE has provided key infrastructure for scientific capacity building, interdisciplinary research, and the development of knowledge-transfer mechanisms linking scientists, students, farmers, advisors, and policy makers. The technical training of professionals, particularly through undergraduate and graduate research projects, has been another key component of the Palo a Pique LTE. This has fostered collaboration with researchers from other institutions, whose expertise has made substantial contributions, for instance, the Global Farm Platform’s researchers from Rothamsted Research.

Dissemination of research findings to producers and advisers plays a key role. An annual results presentation is held, and throughout the year, the experiment receives numerous national and international delegations, including students, producers, technicians, and researchers. All these activities ensure that the ICLS considered in this LTE are locally relevant, economically viable, and environmentally sustainable.

In regions where ICLS have evolved over decades (De Faccio Carvalho et al., 2021), these platforms are essential for assessing how system design and intensification scenarios affect productivity, eco-efficiency, and environmental sustainability. Moreover, the LTE framework represents a replicable model for the co-development, adaptation, and scaling of sustainable ICLS in other temperate regions facing similar intensification challenges.

## Author Biographies

**Fabiana Pereyra-Goday** is an agronomist with an MSc and PhD degrees completed at the Universidad de la República in Uruguay. She currently works as an assistant researcher at the National Institute for Agricultural Research (INIA), specifically on nutrient management and GHG emissions in rice cultivation in Rice-Livestock Systems.

**Walter Ayala** is an agricultural engineer (Universidad de la República, Uruguay), with a PhD in Pastoral Science (Massey University, New Zealand). Expert in plant ecophysiology studies, pasture evaluation, utilization, and persistence. Delegate of the International Rangeland Congress Continuous Committee. Technical Secretariat of “Grupo Campos.” Regional Director—INIA Treinta y Tres, Uruguay.

**Jesús Castillo** is a researcher at INIA in Uruguay and currently leads the Rice–Livestock Systems Research Group. His research focuses on nutrient management and nutrient use efficiency in mixed crop–livestock systems, with particular emphasis on rice and the biogeochemical processes occurring at the dry–flooded soil interface.

**Asma Jebari** is a research scientist focusing on life cycle assessment of agrifood products and has a background in soil organic carbon modeling.

**Graham McAuliffe** is a Reader in Environmental Impact Assessment of Food Systems at Harper Adams University, UK. His research focuses on the sustainability of agri‑food systems, with particular emphasis on Life Cycle Assessment (LCA), particularly environmental and nutritional trade‑offs associated with animal-sourced foods and novel alternatives.

**Michael R.F. Lee** FRSA FRSB FRASE ARAgS, Deputy Vice Chancellor of Harper Adams University. He is an expert in sustainable livestock systems, defining their role in securing global food security at the same time as protecting environmental health (Livestock’s role in human and planetary health).

**Pablo Rovira** is a principal researcher of the Livestock System at INIA Uruguay. He earned his M.Sc. and Ph.D. in Animal Sciences at Colorado State University (USA). His recent work has been focused on beef cattle nutrition and management in integrated crop–livestock systems.

**Taro Takahashi** is a Principal Scientific Officer at Agri-Food & Biosciences Institute, UK. Originally trained as a mathematical economist, his expertise includes bioeconomic modeling and program evaluation of agroecological interventions targeting livestock production systems.

**José A. Terra** (Auburn Univ., USA). Senior Research Agronomist & Soil Scientist at the National Institute of Agricultural Research of Uruguay (INIA). Rice-Livestock Systems Research Program. Estación Experimental del Este, Treinta y Tres, Uruguay. https://orcid.org/0000-0003-0713-9788.

**M. Jordana Rivero** is a researcher in Grazing Livestock Systems and inaugural Chair of the Global Farm Platform. Her work focuses on livestock grazing systems with an emphasis on sustainability, covering pasture and grazing management, livestock performance, animal behavior and welfare, forage quality, and breeding objectives for several livestock species.
